# Transactions of the Medical Society of the State of Pa., May, 1852

**Published:** 1852-11

**Authors:** 


*Transactions of the Medical Society of the State of Pennsylvania, May,* 1852.

No one fact, perhaps, indicates more truthfully the intelligence and progressive spirit of the medical profession, than organization, where the knowledge and experience of each is contributed for the benefit of all. The transactions of our State Medical Societies are invaluable depositories of facts and materials for generalization. The volume before us contains well executed geological maps of several of the counties, an interesting feature, and one which we commend to the consideration of the local societies of our own State.

We congratulate our friends in Pennsylvania upon what they have already accomplished, and shall look with interest for the results of their future labors. J.

